# CD20+ Tumor Infiltrating B Lymphocyte in Oral Squamous Cell Carcinoma: Correlation with Clinicopathologic Characteristics and Heat Shock Protein 70 Expression

**DOI:** 10.1155/2018/4810751

**Published:** 2018-04-04

**Authors:** Nasim Taghavi, Zhaleh Mohsenifar, Alireza Akbarzadeh Baghban, Alireza Arjomandkhah

**Affiliations:** ^1^Oral and Maxillofacial Pathology Department, School of Dentistry, Shahid Beheshti University of Medical Sciences, Tehran, Iran; ^2^Pathology Department, School of Medicine, Shahid Beheshti University of Medical Sciences, Tehran, Iran; ^3^Proteomics Research Center, Department of Basic Science, School of Rehabilitation, Shahid Beheshti University of Medical Sciences, Tehran, Iran

## Abstract

**Objective:**

B lymphocyte infiltration in the tumor microenvironment has been proposed to play pivotal roles in tumor progression. Heat shock protein 70 (HSP70) expressed by tumor cells can induce antitumor immune response. Few studies have examined the clinicopathologic relationship between tumor infiltrating B lymphocyte and HSP70 expression in human cancer. So far, there is no complete knowledge on the relationship in oral squamous cell carcinoma (OSCC). The present study was conducted to evaluate the relationship between tumor infiltrating B lymphocyte and HSP70 expression in OSCC, as well as the clinical outcome.

**Materials and Methods:**

In this retrospective study, the immunohistochemical analysis of 50 OSCC specimens was performed using CD20 and HSP70 antibodies. The relationship between markers' expression and clinicopathologic data was evaluated using Mann–Whitney test, Chi-square test, logistic regression model, and Spearman's correlation coefficient.

**Results:**

The data analysis showed significant correlation between peritumoral CD20^+^ B lymphocyte infiltration and lymph node metastasis (*P* = 0.047). Furthermore, HSP70 expression was significantly correlated with stage (*P* = 0.003), lymph node metastasis (*P* < 0.001), and tumor size (*P* = 0.044). However, no relationship was observed between B lymphocyte infiltration and HSP70 expression.

**Conclusion:**

The results suggest that peritumoral B lymphocyte infiltration and HSP70 expression level have significant association with OSCC and may be considered as prognostic indicators in OSCC. Thus, evaluation of B cells as therapeutic targets in OSCC patients is recommended.

## 1. Introduction

Oral cancer is among the 10 most common malignancies worldwide and the most frequent cancer in south Asian countries. Oral squamous cell carcinoma (OSCC) consists of over 90% of oral cancer characterized by local invasion, aggressive growth pattern, cervical lymph node spread, and high mortality rate [1]. Evidence reveals that oral and pharyngeal cancer accounted for 2.34% of all malignant pathology in Iranian population [2]. Accordingly, squamous cell carcinoma is the most prevalent oral and orofacial cancer [2–5].

B lymphocytes, also known as B cells developed from hematopoietic stem cells, are derived from bone marrow. Mature B lymphocytes constitute 10–20% of circulating peripheral lymphocyte population and function in humoral immune system by producing antibodies. In addition, B cells secrete cytokines, act as antigen presenting cells (APCs), and provide regulatory molecules. B cells recognize antigens through the B cell receptors complex on their cell membrane [6].

B lymphocytes are also a component of tumor infiltrating lymphocytes, although frequently a minor population as compared to T lymphocytes [6, 7]. B cells can identify tumor associated antigens and secrete specific antitumor antibodies [7, 8]. The significant antitumor efficacy of reactive monoclonal antibody in breast carcinoma has been documented [9, 10]. Likewise, there are data showing the features of B cell response such as clonal expansion, somatic mutation, and isotype switching in oral squamous cell carcinoma (OSCC) [11]. Also reported is the increase in B lymphocyte infiltration with the progression of oral epithelium from hyperkeratosis to dysplasia and carcinoma [12], while the prognostic impact of tumor associated B lymphocyte in OSCC has not been fully elucidated.

Heat shock proteins (HSP_s_) are a large family of highly conserved cellular proteins that are classified on the basis of molecular weight, ranging from 15 to 90 KDa [13]. HSP_s_ especially HSP70 are important parts of a protein degradation system and play dual role in tumorigenesis. High levels of HSP70 in cancer cells promote survival and growth of cells by inhibiting apoptosis and unfolding misfolded proteins, in an adenosine triphosphate–dependent fashion. However, tumor derived HSP70 may affect the immunogenicity of tumor cells, deliver them to APCs, and activate antitumor immune response, commonly cellular immune response [14]. The relationship between CD20^+^ B cell infiltration and HSP70 expression in esophageal squamous cell carcinoma proposed the possible antitumor effect of the HSP70-humoral immune system in patients with esophageal cancer [15]. Nevertheless, the correlation of HSP70 expression with tumor infiltrating B lymphocyte in OSCC remains unclear. Therefore, the aim of the present study was to identify the correlation of tumor infiltrating B lymphocyte with important clinicopathologic characteristics and HSP70 expression in OSCC.

## 2. Materials and Methods

### 2.1. Patients

Medical records of patients with OSCC at the Department of Oral Pathology, Shahid Beheshti University of Medical Sciences, Tehran, Iran, between 2010 and 2016 were reviewed. Specimens without complete clinicopathologic data, inadequate paraffin-embedded material, incisional biopsies, and recurrent OSCC were excluded from the study. A total of 50 primary surgically resected OSCC specimens were included in the study. Clinicopathologic data of each case were collected from medical records and by the review of slides. This study was conducted following the ethical criteria of Declaration of Helsinki and was approved by ethics committee of Shahid Beheshti University of Medical Sciences (code number: 95-1258).

### 2.2. Histopathologic Evaluation

Sections of hematoxylin eosin that contain the entire tumor thickness were used to determine the histologic grade. According to WHO classification, specimens were classified into well differentiated, moderately differentiated, and poorly differentiated.

### 2.3. Immunohistochemistry

Three-micrometer thick sections of routinely processed paraffin blocks were prepared. Each section was deparaffinized, rehydrated in xylene, and graded in ethanol and then treated with 3% hydrogen peroxide. For antigen retrieval, the slides were immersed in citrate solution (0.01 Mm, pH = 6.0) and microwaved for 10 minutes. After cooling at room temperature, the slides were incubated with the following primary antibodies: ready to use CD20 mouse monoclonal antibody (Dako, Denmark,) for one hour to detect B cells and HSP70 mouse monoclonal antibody (Novocastra, UK) at a dilution of 1 : 50 for 24 hours. The antibody-antigen complex was visualized with DAB detection kit (k3368; Dako). Tonsil tissue and breast carcinoma were used as positive controls for CD20 and HSP70, respectively.

To evaluate CD20 expression, the mean values of CD20 positive cells in tumor stroma (peritumoral area) and tumor islands (intratumoral area) were separately determined by analyzing the 10 microscopic fields for percentage of positive cells in the most cellular regions at 400x magnification [16]. HSP70 expression was scored using semiquantitative score based on the percentage of positive tumor cells (0–4) and the staining intensity (0–3). The two scores were multiplied, providing a final score as follows: 0, negative; 1–6, low expression; 7–12, high expression [17]. IHC slides were independently examined by two pathologists. Cases with more than 5% deviation were reevaluated together.

### 2.4. Data Analysis

The data were stored and analyzed using SPSS 18 software Package (SSPS, Inc., Chicago, IL, USA). Chi-square test, Spearman's correlation coefficient, and Mann–Whitney test were used to evaluate the relationship between CD20 and HSP70 expression with clinicopathologic variables. Analysis of the expression correlation between HSP70 and CD20 was performed using binary logistic regression model. A *P* value of <0.05 was considered to be statistically significant.

## 3. Results

This retrospective study was carried out on 50 cases of OSCC (20 males and 30 females). The most frequent tumor site was tongue (26%) followed by mandibular alveolar ridge (24%). The average age was 63.3 ± 15.4 years ranging from 30 to 91 years. Tumor size ranged from 0.6 to 8 cm (mean 3.18 ± 1.95). Of the fifty cases, 17 (34%) cases showed lymph node metastasis. Histopathologic grading showed that 31 (62%) cases of OSCC were well differentiated, 13 (26%) cases were moderately differentiated, and 6 (12%) cases were poorly differentiated. In addition, most samples (34%) were in stage I (Table 1).

### 3.1. Relationship of CD20^+^ B Lymphocyte with Clinicopathologic Findings

**Figure 1:**
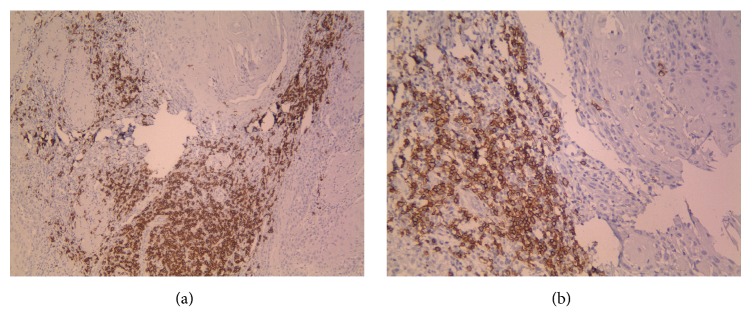
(a, b) represent peritumoral CD20 positive cells in OSCC specimens (×100, ×200).

**Figure 2:**
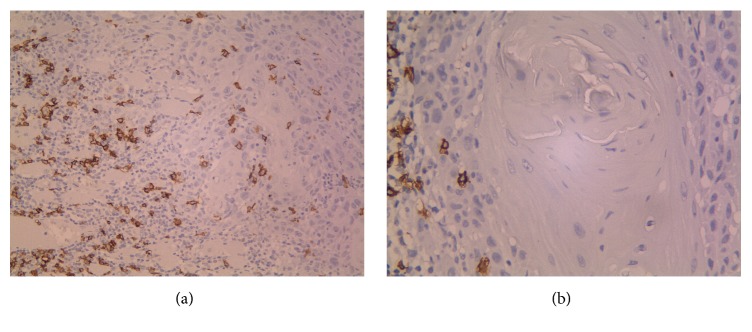
(a, b) represent intratumoral CD20 positive cells in OSCC specimens (×200, ×400).

The number of CD20^+^ B cells in peritumoral and intratumoral areas ranged between 0 and 120 (mean 24.54) and 0 and 32 (mean 2.68), respectively (Figures 1 and 2).

Data analysis showed significant inverse correlation between the mean number of peritumoral CD20^+^ B lymphocyte and lymph node metastasis (*P* = 0.047). Furthermore, a relationship was found between the mean number of intratumoral CD20^+^ B lymphocyte and gender (*P* = 0.013) as it was higher in males. No correlation was found between CD20^+^ B lymphocyte with stage and tumor size (*P* > 0.05) (Table 2).

### 3.2. Relationship between HSP70 Expression and Clinicopathologic Findings

**Figure 3:**
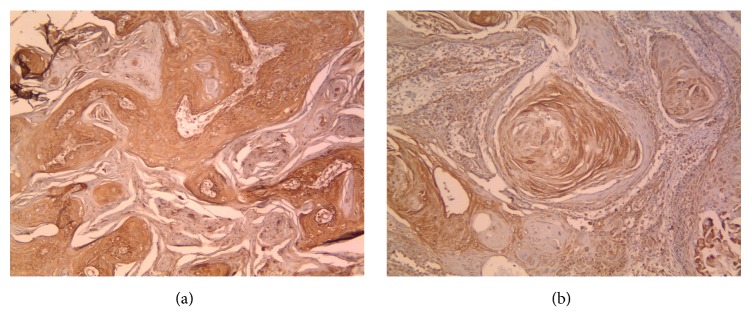
(a, b) represent cytoplasmic HSP70 expression in OSCC specimens (×100).

Of the 50 OSCC cases, 21 (42%) cases were identified as low expression and 29 (58%) cases as high expression (Figure 3). The findings showed significant correlation between HSP70 expression and tumor size (*P* = 0.44). The odds ratio (OR) was 1/4. Chi-square test showed significant correlation between HSP70 expression and lymph node metastasis (*P* < 0.001) since 84.8% of nonmetastatic cases expressed high HSP70 whereas 94.1% of the metastatic cases presented low expression of HSP70. A significant correlation was also found between HSP70 expression and stage (*P* = 0.003). However, the correlation between HSP70 and histologic grade was not significant (*P* = 0.058) (Table 3).

### 3.3. Association of CD20^+^ B Lymphocyte Infiltration and HSP70 Expression

There was no relationship for binary logistic regression model between the mean number of intratumoral and peritumoral B lymphocytes with HSP70 expression, respectively (*P* = 0.18, *P* = 0.771).

## 4. Discussion

The global incidence of oral cancer is increasing, particularly in women and younger population owing to changes in life style. Despite the recent advanced treatment options including radical surgery, chemotherapy, and radiotherapy in various combinations, the prognosis remains poor. Studies conducted over the past 2 decades have established clinical stage, lymph node metastasis, extracapsular spread, and pattern of invasion as superior clinicopathologic prognostic indicators in OSCC patients [13, 18, 19].

Immune cells infiltration into the tumor microenvironment and their positive prognostic relevance have been described in some solid tumors. In recent years, literature has focused on the antitumor activity of T lymphocyte, especially, CD8^+^ T lymphocyte [20–22]. The role of B cells as the main component of the humoral immune system has been addressed in few investigations with conflicting results [23–25]. With regard to neoplasms, there are proofs that support HSP70 and anti-HSP70 antibody importance in immune function regulation during tumorigenesis [13, 15].

This is the first study to examine the relationship between CD20^+^ tumor infiltrating B lymphocyte and clinical outcome in OSCC. The most striking result was the significant inverse correlation of peritumoral CD20^+^ B cells with lymph node metastasis which is in agreement with reports on non-small-cell lung cancer, esophageal cancer, and breast cancer. These studies emphasize that the presence of higher CD20^+^ B cells in tumor microenvironment is in relation to a favorable prognosis and survival rate [7, 12, 26–28]. A decreasing trend was also reported for CD20+ cell infiltration in less differentiated OSCC [29]. On the other hand, Lundgren et al. [24] did not find any correlation between CD20^+^ B cells infiltration and prognosis in ovarian epithelial tumors. Distel et al. indicated that higher number of CD20^+^ B cells in early stage of hypopharynx squamous cell carcinoma was associated with improved locoregional control. In advanced tumor, CD20^+^B cell infiltration was a negative prognostic factor [25]. They suggested that CD20^+^B cells play an important antitumor role in early stage of tumorigenesis by antigen presentation and antibody production.

Overall assessment of the recently reported investigation shows the dual role of defense and offense for B lymphocyte in the tumor microenvironment. The activated B cells can inhibit tumor proliferation using antibody production through IG2b dependent pathway which is highly cytotoxic toward tumor cells. Of interest, B cells stimulated by IL-21 are able to kill tumor cells by producing granzyme B. B cells also facilitate CD4^+^ T cell memory function and CD8^+^ T cells proliferation. High activity of B cells and better survival rate following treatment with IL-12 in head and neck squamous cell carcinoma have also been reported. On the other hand, B cells produce IL-10 and TGF-B which inhibit Th_1_/CD8+ T cells function and promote tumor proliferation [6, 7, 30–32]. It seems that the status of B cells in different contexts is crucial, as cellular immune response is facilitated by activated B cells but inhibited by resting B cells. B cells are usually activated in human cancers, inducing the possibility of positive role in tumor immunity [31].

In the current study, the second important finding was the significant association of HSP70 expression with stage and lymph node metastasis. The results are in keeping with earlier findings by Nakajima et al. [15] and Tavassol et al. [33] and disagreed with the reported data on non-small-cell lung carcinoma, breast cancer, and melanoma [34–36]. In line with the Deyhimi and Azmoudeh study [37], the analysis of the present study revealed no significant association between HSP70 expression and histologic grade. No association was found between CD20^+^ B cell infiltration and HSP70 expression in the series of the present study which is inconsistent with Nakajima et al. study in esophageal squamous cell carcinoma [15].

HSP70 expressed at low levels in normal physiologic conditions is regarded as a molecular chaperon. Given reports demonstrated altered expression level of HSP70 in malignancies. Overexpression of HSP70 can inhibit external and internal apoptosis pathway by binding to BAX and death receptor DR4 and DR5, but it plays the role of a tumor specific antigen which is highly immunogenic. It promotes cell surface antigen peptides presentation using overexpression of MHC class I and activates antitumor innate and adaptive immune response [13]. Of interest, HSP70 cellular localization has important role in mediating immunological function since extracellularly located HSPs act as a powerful cytokine, eliciting immunocompetent cells. HSP70 also stimulates a proinflammatory signal transduction that results in upregulation of TNF-*α* and IL-6 via CD-14 dependent pathway. The impact of HSP70 expression on tumor immunogenicity may be related to variable functions of CD91 molecule (HSP70 receptor). The complex of HSP70-CD91 may eclipse apoptotic cell phagocytosis and induce more marked Th1/CD8 immune response [33, 35].

It is of great importance that the tumor microenvironment contents (pH, oxygen), oncoproteins made during tumorigenesis, and genetic alterations may change the HSP70 function and context. In fact, HSP70 function and response may be organ or tumor specific because of the HSP70 associated proteins within a unique molecular milieu [20, 33]. Notably, rational use in therapeutic strategies is suggested for HSP70 due to its different and paradoxical role in solid tumors which are affected by numerous factors. To examine the correlation of HSP70 expression with CD20^+^ B cell infiltration and possible mechanism, further molecular and biologic studies are required.

## 5. Conclusion

The results suggest that peritumoral B lymphocyte infiltration and HSP70 expression level have significant association with OSCC and may be used as prognostic indicators in OSCC. No relationship was found between B lymphocyte infiltration and HSP70 expression.

## Figures and Tables

**Table 1:** Clinicopathologic characteristics in OSCC patients.

Parameter	Number (%)
Sex	
Female	20 (40%)
Male	30 (60%)
Age (mean ± SD)	63.3 ± 15.4
Location	
Tongue	13 (26%)
Mandibular alveolar ridge	12 (24%)
Buccal mucosa	8 (16%)
Floor of mouth	7 (14%)
Palate	4 (8%)
Maxillary alveolar ridge	3 (6%)
Lower lip	3 (6%)
Grade	
Well differentiated	31 (62%)
Moderately differentiated	13 (26%)
Poorly differentiated	6 (12%)
Stage	
I	17 (34%)
II	11 (22%)
III	13 (26%)
IV	9 (18%)
Lymph node metastasis	
Yes	17 (34%)
No	33 (66%)
Tumor size	
<2 cm	16 (32%)
Between 2 and 4 cm	20 (40%)
>4 cm	14 (28%)

**Table 2:** Relationship between clinicopathologic characteristics and CD20 expression in OSCC patients.

Parameter	Intratumoral CD20	Peritumoral CD20
Size	*r* = 0.027	*R* = −0.63
	*P* = 0.854^†^	*P* = 0.665^†^
Stage	*r* = −0.090	*r* = −0.248
	*P* = 0.535^†^	*P* = 0.083^†^
Grade	*r* = −0.052	*r* = 0.153
	*P* = 0.717^†^	*P* = 0.290^†^
Lymph node metastasis	*P* = 0.051^1^	*P* = 0.047^1^
Gender	*P* = 0.013^1^	*P* = 0.112^1^

†: based on Spearman correlation coefficient; 1: based on binary logistic regression model; bolded values are statistically significant.

**Table 3:** Relationship between clinicopathologic characteristics and HSP70 expression in OSCC patients.

Parameter	Number (%)	Low expression	High expression	*P* value
Grade				
Well differentiated	31 (62%)	7 (22.6%)	24 (77.4%)	0.058
Moderately differentiated	13 (26%)	8 (61.5%)	5 (38.5%)	
Poorly differentiated	6 (12%)	6 (100%)	0 (0%)	
Stage				
I	17 (34%)	2 (11.8%)	15 (88.2%)	**0.003**
II	11 (22%)	3 (27.3%)	8 (72.7%)	
III	13 (26%)	11 (84.6%)	2 (15.4%)	
IV	9 (18%)	5 (55.6%)	4 (44.4%)	
Lymph node metastasis				
Yes	17 (34%)	16 (94.1%)	1 (5.9%)	**<0.001**
No	33 (66%)	5 (15.2%)	28 (88.4%)	
Tumor size				
<2 cm	16 (32%)	2 (12.5%)	14 (87.5%)	**0.044**
Between 2 and 4 cm	20 (40%)	11 (55%)	9 (45%)	
>4 cm	14 (28%)	8 (57.1%)	6 (49.2%)	

Bolded values are statistically significant.
